# Association Between Time–Averaged Serum Uric Acid Level and Subsequent Renal Involvement in Premenopausal Patients with Systemic Lupus Erythematosus

**DOI:** 10.5152/ArchRheumatol.2025.11144

**Published:** 2025-12-01

**Authors:** Su Jin Choi, Min Wook So, Kyung Don Yoo, Sunggun Lee, Seung Won Choi, Doo-Ho Lim

**Affiliations:** 1 Division of Rheumatology, Department of Internal Medicine, Ulsan University Hospital, University of Ulsan College of Medicine, Ulsan, South Korea; 2 Division of Rheumatology, Department of Internal Medicine, Pusan National University Yangsan Hospital, Pusan National University School of Medicine, Yangsan, South Korea; 3 Division of Nephrology, Department of Internal Medicine, Ulsan University Hospital, University of Ulsan College of Medicine, Ulsan, South Korea; 4 Department of Internal Medicine, Haeundae Paik Hospital, Inje University College of Medicine, Busan, South Korea

**Keywords:** Lupus nephritis, premenopause, systemic lupus erythematosus, uric acid

## Abstract

**Background/Aims:**

Serum uric acid (SUA) is a potential risk factor for renal involvement in systemic lupus erythematosus (SLE). However, the impact of cumulative SUA levels on subsequent renal involvement in premenopausal women remains unclear. The association between cumulative SUA levels and the development of subsequent renal involvement in premenopausal patients with SLE was evaluated.

**Materials and Methods:**

A retrospective review of 112 premenopausal women with newly diagnosed SLE, and no renal involvement at diagnosis was conducted in a tertiary medical center. Clinical characteristics at diagnosis and during follow-up were compared between patients who developed subsequent renal involvement and those who did not. Time-averaged SUA (TA-SUA) was calculated from the area under the curve during follow-up. Cox proportional hazards analysis was performed to evaluate the factors associated with subsequent renal involvement.

**Results:**

The median TA-SUA level among 112 patients was 4.2 mg/dL (IQR, 3.8-4.8). During a median follow-up of 4.4 years, 25 patients developed subsequent renal involvement. The proportion of patients with TA-SUA level ≥4.2 mg/dL was higher in patients with subsequent renal involvement than in those without (84.0% vs. 46.0%, *P* = .001). In multivariate Cox analysis, TA-SUA level ≥4.2 mg/dL was associated with a higher risk of subsequent renal involvement (hazard ratio 6.389, *P* = .014). Systemic lupus erythematosus disease activity index score and the presence of anti-Smith antibodies at diagnosis were also associated with subsequent renal involvement.

**Conclusion:**

High TA-SUA levels are independently associated with an increased risk of subsequent renal involvement in premenopausal patients with SLE, underscoring the importance of cumulative SUA levels.

**Main Points**

- Elevated time-averaged serum uric acid levels were independently associated with an increased risk of developing renal involvement in premenopausal patients with systemic lupus erythematosus.
- Baseline disease activity and the presence of anti-Smith antibodies were also identified as significant predictors of subsequent renal involvement in systemic lupus erythematosus.
- Long-term monitoring of serum uric acld levels may aid in predicting renal involvement in systemic lupus erythematosus.

## Introduction

Systemic lupus erythematosus (SLE) is a chronic autoimmune disease that affects multiple organ systems. Renal involvement in SLE, known as lupus nephritis, is a major organ complication and occurs in up to 60% of patients with SLE.^1,2^ It significantly impacts morbidity and mortality in SLE, with approximately 20% of patients progressing to end-stage renal disease.^3^ Therefore, early detection and treatment of renal involvement are important to achieve better outcomes.

Several factors associated with renal involvement in SLE have been established, including elevated anti-double-stranded DNA antibody (anti-dsDNA) titer and low serum C3 and C4 levels.^4^ However, as the pathogenesis of renal involvement is multifactorial, the factors involved are yet to be fully elucidated. Serum uric acid (SUA) has been suggested as a potential factor for the development of renal involvement in SLE. Uric acid, an end product of purine metabolism in humans, is primarily produced in the liver and excreted through the kidneys and intestines. Elevated SUA levels have pro-inflammatory and pro-oxidant properties and are associated with various diseases, such as gout, hypertension, coronary heart disease, stroke, and chronic kidney disease.^5^ Hyperuricemia is more commonly observed in patients with SLE than in the general population and is associated with disease activity and overall damage.^6,7^ Previous studies have also found an association between SUA levels and various aspects of renal involvement, including the development of renal involvement, progression to chronic kidney disease, and long-term renal outcomes.^8-10^ However, this association has not been consistently reported.^11^

Understanding the relationship between SUA levels and the risk of developing renal involvement is crucial for the early identification and control of at-risk patients. Renal involvement in SLE typically develops within the first 5 years after diagnosis.^12^ However, it is not always present at the time of diagnosis and may develop later in the disease course. Uric acid has been linked to kidney damage through inflammatory and oxidative processes, and SUA levels can fluctuate over time. Considering the long-term nature of SLE, a single-point measurement may not adequately represent long-term exposure to uric acid. To better reflect this cumulative burden, the concept of time-averaged or cumulative SUA has been used in various chronic diseases, including myocardial infarction, stroke, diabetes, IgA nephropathy, and in patients undergoing peritoneal dialysis.^13-17^ Despite this, few studies have evaluated the cumulative effect of SUA levels on subsequent renal involvement over the disease course of SLE. Additionally, SUA levels are influenced by age and sex, and SLE primarily occurs in women of childbearing age. However, studies focusing on this specific population are lacking. Therefore, this study aimed to evaluate factors, including SUA levels, associated with the development of subsequent renal involvement in premenopausal patients with SLE.

## Methods

### Patients

This study enrolled premenopausal patients with newly diagnosed SLE at a tertiary referral hospital in Korea between June 2000 and September 2022. All patients met the 1997 American College of Rheumatology (ACR) criteria for the classification of SLE. Since postmenopausal alterations in female hormone levels are associated with elevated SUA levels, this study focused on premenopausal patients who were aged between 16 and 45 years.^18^ To investigate the factors associated with the development of subsequent renal involvement during follow-up period, patients who had renal involvement at the time of SLE diagnosis were excluded from this study. Additional exclusion criteria included active infection, malignancy, other renal diseases such as IgA nephropathy, or missing SUA data.

This study was conducted in accordance with the principles of the Declaration of Helsinki. The Institutional Review Board of Ulsan University Hospital, Ulsan, South Korea approved the study (IRB number: 2024-07-039, date: 12.08.2024) and waived the requirement for informed consent due to its retrospective design.

### Data Collection

The electronic medical records of the study patients were retrospectively reviewed. Baseline data were collected at the time of SLE diagnosis and included age; presence of hypertension and diabetes mellitus; body mass index; SLE Disease Activity Index-2K (SLEDAI-2K) score; estimated glomerular filtration rate (eGFR); serum creatinine, serum albumin, SUA, serum C3, and serum C4 levels; anti-dsDNA titer; and presence of anti-extractable nuclear antigen antibodies. Hyperuricemia was defined as SUA levels greater than 6 mg/dL.^19^ The SUA levels were also assessed at each visit during the follow-up period. Time-averaged SUA (TA-SUA) was calculated using the trapezoidal rule by dividing the area under curve for SUA during the follow-up period by the follow-up duration.^20^ Patients were classified into 2 groups based on the median TA-SUA level. The last SUA level was defined as the SUA level obtained at the last follow-up date. Medications prescribed during the follow-up period, including hydroxychloroquine, immunosuppressants (cyclophosphamide, mycophenolate mofetil, azathioprine, and tacrolimus), glucocorticoids, aspirin, and urate-lowering agents, were reviewed.

According to the 2019 European League Against Rheumatism (EULAR) and ACR classification criteria for SLE,^21^ renal involvement was defined as a proteinuria level of >500 mg/day (or equivalent spot urine protein-to-creatinine ratio >500 mg/g) and/or biopsy-proven lupus nephritis. During follow-up, the development of subsequent renal involvement in patients was assessed. The last follow-up date was defined as the date of diagnosis of subsequent renal involvement, the date of last visit to the hospital, or the date on which a patient turned 45 years.

### Statistical Analysis

Continuous variables are presented as median (interquartile range [IQR]) and were assessed using the Mann–Whitney *U* test. Categorical data are expressed as numbers (percentages) and were analyzed using the chi-squared and Fisher’s exact tests. Kaplan–Meier analysis with the log-rank test was performed to evaluate the cumulative incidence of subsequent renal involvement according to TA-SUA levels. Univariate and multivariate Cox proportional hazards analyses were performed to calculate the hazard ratio (HR) and 95% CI for the risk associated with subsequent renal involvement. Variables with a *P*-value <0.2 in the univariate Cox proportional hazards analysis were included in the multivariate analysis. A *P-*value <.05 was considered statistically significant in all other analyses. A post hoc power analysis was conducted to evaluate the adequacy of the sample size, focusing on the association between TA-SUA level (≥4.2 mg/dL) and subsequent renal involvement. Power estimates for the Pearson’s chi-square test, Cox proportional hazards model, and log-rank test were 0.920, 0.982, and 0.965, respectively, indicating sufficient power for the primary analyses. The data analyses were conducted using R version 4.4.1 (R Foundation; Vienna, Austria).

## Results

### Characteristics of Premenopausal Systemic Lupus Erythematosus Patients with and Without Subsequent Renal Involvement

A total of 160 premenopausal women with newly diagnosed SLE were identified. Among them, 48 patients had renal involvement at the time of SLE diagnosis. Consequently, the remaining 112 patients were followed up for a median period of 4.4 years (IQR, 2.0-8.0). During follow-up period, 25 (22.3%) patients developed subsequent renal involvement, with a median time from SLE diagnosis of 3.1 years (IQR, 1.1-7.4).

Table 1 summarizes the characteristics of patients with and without subsequent renal involvement. At baseline, patients who developed subsequent renal involvement were younger and had higher SLEDAI-2K scores, lower serum albumin levels, lower serum C3 levels, lower serum C4 levels, higher anti-dsDNA titers, and a higher presence of anti-Smith antibodies than those who did not develop subsequent renal involvement. No significant differences existed in hypertension, diabetes mellitus, body mass index, and eGFR between the 2 groups. During the follow-up, age at the last follow-up date was lower in patients with subsequent renal involvement than in those without subsequent renal involvement. No significant differences were found between the 2 groups in the use of medications, including hydroxychloroquine, immunosuppressants, glucocorticoids, and aspirin. Neither group included patients on urate-lowering agents, such as febuxostat or allopurinol, during the follow-up period.

### Association Between Serum Uric Acid Level and Subsequent Renal Involvement

The median baseline SUA, TA-SUA, and last SUA levels in the overall SLE patients were 4.2 mg/dL (IQR, 3.5-5.0), 4.2 mg/dL (IQR, 3.8-4.8), and 4.3 mg/dL (IQR, 3.4-4.8), respectively. While baseline SUA levels did not differ significantly between patients with and without subsequent renal involvement, TA-SUA (4.5 mg/dL [IQR, 4.2-5.5] vs. 4.1 mg/dL [IQR, 3.6-4.7], *P* = .005) and last SUA levels (4.7 mg/dL [IQR, 4.4-5.9] vs. 4.0 mg/dL [IQR, 3.4-4.6], *P* < .001) were higher in patients with subsequent renal involvement than in those without subsequent renal involvement (Table 1). The proportion of patients with a TA-SUA level ≥4.2 mg/dL was higher in those with subsequent renal involvement than in those without subsequent renal involvement (84.0% vs. 46.0%, *P* = .001).

### Time–Averaged Serum Uric Acid Level as a Risk Factor Associated with Subsequent Renal Involvement

We identified the factors associated with subsequent renal involvement during the follow-up period (Table 2). In the univariate Cox proportional hazards analysis, the baseline characteristics that were significantly associated with subsequent renal involvement included age, SLEDAI-2K scores, serum albumin levels, serum C3 levels, serum C4 levels, and the presence of anti-Smith antibodies. A TA-SUA level ≥ 4.2 mg/dL and last SUA levels were also associated with an increased risk of subsequent renal involvement. The results of the multivariate analysis indicated that higher SLEDAI-2K score (HR = 1.097; 95% CI, 1.021-1.180; *P* = .012) and the presence of anti-Smith antibodies (HR = 2.533; 95% CI, 1.040-6.167; *P* = .041) at SLE diagnosis were associated with the development of subsequent renal involvement. A TA-SUA level ≥4.2 mg/dL (HR = 6.389; 95% CI, 1.457-28.024; *P* = .014) was also a significant independent factor associated with subsequent renal involvement.

**Figure 1.**
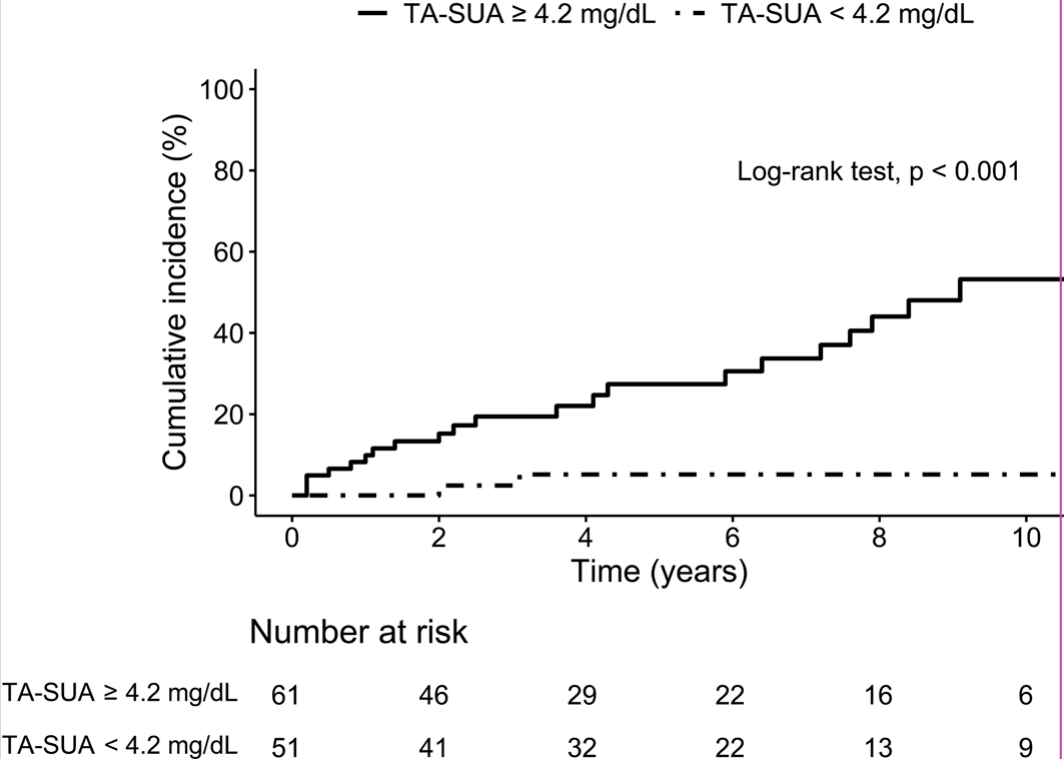
Kaplan–Meier curve for subsequent renal involvement in premenopausal patients with SLE according to time-averaged serum uric acid levels. SLE, systemic lupus erythematosus; TA-SUA, time-averaged serum uric acid.

Figure 1 shows the Kaplan–Meier curves for subsequent renal involvement in patients without baseline renal involvement. The overall cumulative incidence of subsequent renal involvement was 17.2% at 5 years and 34.0% at 10 years. Patients with a TA-SUA level ≥4.2 mg/dL had cumulative incidence rates of 27.4% at 5 years and 53.2% at 10 years, while those with a TA-SUA level <4.2 mg/dL consistently showed rates of 5.1% at both 5 and 10 years. The log-rank test indicated a significantly higher incidence of subsequent renal involvement in patients with a TA-SUA level ≥4.2 mg/dL than in those with a TA-SUA level <4.2 mg/dL (*P* < .001).

## Discussion

This study investigated the relationship between TA-SUA levels and the development of subsequent renal involvement in premenopausal women with SLE. To minimize the effect of sex and age on SUA levels, only premenopausal women were included. The study demonstrated that higher TA-SUA levels were associated with an increased risk of subsequent renal involvement in premenopausal patients with SLE. These findings highlight the importance of cumulative SUA levels in the development of renal involvement during the disease course.

Previous studies reported the association between SUA levels and the development of renal involvement in SLE. Yang et al^8^ found that high SUA levels were independently associated with the onset of lupus nephritis, with an optimal cut-off value of 330 μmol/L. Another prospective study revealed that high baseline SUA levels were associated with an increased risk of new renal damage incidence, with an HR of 3.21.^22^ Uric acid plays a crucial role in the pathological mechanisms of kidney damage. It contributes to the occurrence of kidney injury by inducing inflammation and promoting vascular remodeling. Acting as a damage-associated molecular pattern molecule, uric acid activates the NLRP3 inflammasome and interleukin-1β through the Toll-like receptors on renal proximal tubular cells.^23^ High SUA levels promote the infiltration of T cells and macrophages and upregulate the expression of MPC-1, NF–κB, MAPK, and C-reactive protein.^24,25^ Additionally, uric acid generates reactive oxygen species (ROS), inhibits nitric oxide synthesis, and promotes oxidative stress, mitochondrial damage, and apoptosis in renal tubular epithelial cells, consequently exacerbating tissue damage and inflammation. It also activates the renin-angiotensin system, leading to renal vasoconstriction, endothelial dysfunction, and vascular smooth muscle proliferation.^26^ These processes contribute to vascular remodeling, resulting in glomerulosclerosis and interstitial fibrosis.^27^ In a study of patients with lupus nephritis, those with high SUA levels compared with those with low SUA levels exhibited more crescents, a higher degree of mesangial matrix, increased endothelial cell proliferation, and more inflammatory cell infiltration.^28^

The study demonstrated the impact of TA-SUA on the development of subsequent renal involvement in patients with SLE. The proportion of patients with a TA-SUA level ≥4.2 mg/dL was 84.0% in those with subsequent renal involvement compared with 46.0% in those without subsequent renal involvement. The 10-year cumulative incidence of subsequent renal involvement was 53.2% in patients with a TA-SUA level ≥4.2 mg/dL but only 5.1% in patients with a TA-SUA level <4.2 mg/dL. Cox proportional hazards analysis revealed that the risk of subsequent renal involvement in patients with a TA-SUA level ≥ 4.2 mg/dL was 6.389 times higher than that of patients with a TA-SUA level < 4.2 mg/dL. These results indicate that the cumulative burden of SUA may contribute to the development of subsequent renal involvement. Interestingly, the baseline SUA level did not have a statistically significant influence on the risk of developing subsequent renal involvement in patients with SLE. Although the last SUA levels were higher in patients with subsequent renal involvement than in those without subsequent renal involvement, they were not an independent factor influencing this risk in the Cox proportional hazards analysis. These findings suggest that persistently high SUA levels, rather than a single elevated measurement, may be more critical for the development of subsequent renal involvement in patients with SLE. These findings also indicate that uric acid may be an independent factor contributing to kidney injury rather than resulting from existing kidney damage.

Notably, this association is observed when considering only premenopausal women, who typically have lower SUA levels than those had by other populations. Normal SUA levels in premenopausal women typically range from 2.6 to 6.0 mg/dL, and the prevalence of hyperuricemia is estimated at approximately 5% or less in Korean women aged 19 to 49 years.^19 ,29^ The SUA levels are generally lower in premenopausal women than in men and postmenopausal women, primarily due to the uricosuric effect of estrogen, which enhances the renal excretion of uric acid.^30^ Age-related changes in metabolic processes affect SUA levels across different age groups. A previous study on premenopausal patients with SLE reported that 64% of patients with high SUA levels had renal involvement, compared with only 16% of patients with normal SUA levels.^7^ The study also showed the association between TA-SUA and subsequent renal involvement in premenopausal women. The median TA-SUA level was 4.5 mg/dL and 4.1 mg/dL in patients with and without subsequent renal involvement, respectively. Although the median TA-SUA levels in both groups were within the normal range and the numerical difference between the groups was small, a statistically significant difference was observed. Previous research has shown that soluble uric acid levels that are as low as 5 mg/dL can induce the NLRP3 inflammasome in macrophages and increase interleukin-1β production.^31^ Given that TA-SUA is a cumulative value and that this study assessed the data of premenopausal women, a small difference in TA-SUA levels, sustained over a long period, might have a significant impact.

Sex difference in the effects of uric acid on the kidneys is not yet fully understood. A study that examined renal prognosis according to SUA levels in patients with lupus nephritis found that SUA was an independent risk factor for the lupus nephritis progression in women but not in men.^11^ A similar result was observed in another observational study in Southern China.^32^ Although the mechanisms behind these sex differences are not yet clear, women may possibly be more susceptible to the effects of uric acid than that observed in men. Kohagura et al^33^ reported that the cut-off value of SUA associated with renal arteriolar hyalinosis was significantly lower in women (≥5 mg/dL) than in men (≥7 mg/dL). In a study on the impact of plasma xanthine oxidoreductase (XOR) activity, which is linked to ROS production, women had a lower plasma XOR activity cut-off value for predicting the occurrence of coronary artery spasm than that had by men.^34^ Despite this assumption, the differences in the effect of uric acid between sexes remains unclear, highlighting the need for further studies to better understand these differences.

In the multivariate Cox proportional hazards analysis, a high SLEDAI score at the time of SLE diagnosis was identified as a significant predictor of subsequent renal involvement. The SLEDAI score includes components associated with renal involvement, including hematologic manifestation, serositis, complement levels, and anti-dsDNA titers.^35,36^ This finding suggests that high disease activity at diagnosis may increase the risk of developing renal involvement later. Moreover, the presence of anti-Smith antibodies was also associated with the development of subsequent renal involvement. Anti-Smith antibodies are considered potential contributors to lupus nephritis,^37^ and a previous study suggested a link between these antibodies and the genetic predisposition to lupus nephritis.^38^ Consistent with these findings, this study indicated that a high SLEDAI score and the presence of anti-Smith antibodies at SLE diagnosis can independently predict subsequent renal involvement in patients with SLE.

This study has several limitations. First, some patients with renal involvement did not undergo kidney biopsy, which is the gold standard for diagnosing lupus nephritis. To address this limitation, renal involvement was defined in this study based on the 2019 EULAR/ACR classification criteria for SLE, and patients with suspected or diagnosed with other renal diseases were excluded. Second, additional confounding factors, such as diet, alcohol consumption, physical activity, or other comorbidities, could have affected uric acid levels; however, information on these variables was not available. Third, this was a retrospective single-center study. Further research with larger patient populations and a prospective design is needed. Nevertheless, the strength of this study lies in its focus on the impact of TA-SUA level on the development of future renal involvement in SLE. Notably, this association was observed in premenopausal patients with SLE, allowing this study to control for the influence of age and sex on SUA levels.

This study demonstrated that TA-SUA level was an independent factor for the development of subsequent renal involvement in premenopausal patients with SLE, even after adjusting for known risk factors such as disease activity and serological markers. In contrast, neither the baseline nor the last SUA level independently predicted this risk. While most previous studies have focused on the association between single-point SUA measurements and renal involvement, this study suggests that sustained uric acid levels, as reflected by TA-SUA, may serve as a more reliable marker of renal involvement. This approach allows for better risk estimation and suggests a potential contribution of cumulative SUA exposure to the development of subsequent renal involvement in SLE. These findings highlight the potential value of longitudinal SUA monitoring in patients with SLE.

## Figures and Tables

**Table 1.** Characteristics of Premenopausal SLE Patients with or Without Subsequent Renal Involvement

Variable	Subsequent Renal Involvement (n = 25)	No Subsequent Renal Involvement (n = 87)	*P*
Baseline characteristics			
Age at SLE diagnosis, years	22 (20-31)	34 (27-39)	<.001
Hypertension	0 (0%)	2 (2.3%)	1.000
Diabetes mellitus	0 (0%)	1 (1.1%)	1.000
Body mass index, kg/m^2^	21.1 (20.1-22.9)	20.8 (19.1-22.7)	.514
SLEDAI-2K score	11 (8-16)	8 (5-10)	.001
Serum creatinine, mg/dL	0.70 (0.55-0.80)	0.66 (0.59-0.81)	.837
eGFR, mL/min/1.73m^2^	120.2 (102.0-129.1)	114.0 (94.7-121.0)	.068
Serum albumin, mg/dL	3.6 (3.3-4.1)	4.2 (3.8-4.5)	.001
Baseline SUA, mg/dL	4.5 (3.7-5.4)	4.0 (3.3-4.9)	.151
Hyperuricemia	4 (16.0%)	6 (6.9%)	.226
Serum C3, mg/dL	51.2 (34.2-77.5)	79.9 (57.4-96.7)	.003
Serum C4, mg/dL	5.9 (4.4-11.4)	13.9 (8.8-20.1)	.004
Anti-dsDNA titer, IU/mL	56.0 (15.3-150.0)	10.0 (2.6-39.0)	.003
Anti-ENA positivity			
Anti-Ro	14/23 (60.9%)	53/82 (64.6%)	.740
Anti-La	6/23 (26.1%)	28/80 (35.0%)	.423
Anti-Smith	15/23 (65.2%)	25/75 (33.3%)	.006
Anti-RNP	13/24 (54.2%)	37/75 (49.3%)	.680
Follow-up characteristics			
Follow-up duration, years	3.1 (1.1-7.4)	4.8 (2.3-8.4)	.081
Age at last follow-up date, years	29 (23-37)	40 (34-44)	<.001
TA-SUA, mg/dL	4.5 (4.2-5.5)	4.1 (3.6-4.7)	.005
TA-SUA ≥4.2 mg/dL	21 (84.0%)	40 (46.0%)	.001
Last SUA, mg/dL	4.7 (4.4-5.9)	4.0 (3.4-4.6)	<.001
Medication			
Hydroxychloroquine	24 (96.0%)	85 (97.7%)	.535
Immunosuppressants	10 (40.0%)	20 (23.0%)	.090
Cumulative prednisolone dose, g	5.1 (2.6-17.5)	3.5 (1.3-7.5)	.056
Aspirin	2 (8.0%)	14 (16.1%)	.517

Data are expressed as median (interquartile range) or number (%).

Anti-dsDNA, anti-double-stranded DNA antibody; Anti-ENA, anti-extractable nuclear antigen antibody; eGFR, estimated glomerular filtration rate; SLE, systemic lupus erythematosus; SLEDAI-2K, SLE disease activity index-2K; SUA, serum uric acid; TA-SUA, time-averaged serum uric acid.

**Table 2.** Cox Proportional Hazards Analysis of Risk Factors Associated with Subsequent Renal Involvement in Premenopausal Patients with SLE

Variable	Univariate Analysis	*P*	Multivariate Analysis	*P*
Baseline characteristics				
Age at SLE diagnosis, years	0.926 (0.872-0.984)	.013		
SLEDAI-2K score	1.141 (1.061-1.227)	<.001	1.097 (1.021-1.180)	.012
eGFR, mL/min/1.73m^2^	1.020 (0.994-1.047)	.132		
Serum albumin, mg/dL	0.366 (0.191-0.702)	.002		
Baseline SUA, mg/dL	1.255 (0.894-1.763)	.190		
Serum C3, mg/dL	0.984 (0.971-0.997)	.004		
Serum C4, mg/dL	0.933 (0.882-0.987)	.016		
Anti-dsDNA titer, IU/mL	1.002 (1.000-1.005)	.088		
Anti-Smith positivity	3.193 (1.337-7.626)	.009	2.533 (1.040-6.167)	.041
Follow-up characteristics				
TA-SUA ≥4.2 mg/dL	8.437 (1.963-36.266)	.004	6.389 (1.457-28.024)	.014
Last SUA, mg/dL	1.649 (1.156-2.353)	.006		
Hydroxychloroquine	0.273 (0.036-2.072)	.209		
Immunosuppressants	1.847 (0.785-4.347)	.160		
Cumulative prednisolone dose, g	1.020 (0.975-1.067)	.397		
Aspirin	0.195 (0.026-1.464)	.112		

Anti-dsDNA, anti-double-stranded DNA antibody; eGFR, estimated glomerular filtration rate; SLE, systemic lupus erythematosus; SLEDAI-2K, SLE Disease Activity Index-2K; SUA, serum uric acid; TA-SUA, time-averaged serum uric acid.
